# Effects of homocysteine on nonalcoholic fatty liver related disease: A mendelian randomization study

**DOI:** 10.3389/fmolb.2022.1083855

**Published:** 2022-12-06

**Authors:** Pengcheng Chen, Ze Yang, Lingyun Guo, Yingfei Huang, Jingjia Li, Xin Chen

**Affiliations:** ^1^ School of Electronics and Information Engineering, Institute of Big Data and Artificial Intelligence in Medicine, Taizhou University, Taizhou, China; ^2^ Department of Biochemistry and Molecular Biology, School of Medicine, Zhejiang University, Hangzhou, Zhejiang, China; ^3^ Department of Biochemistry and Cancer Medicine, International Institutes of Medicine, The Fourth Affiliated Hospital of Zhejiang University School of Medicine, Yiwu, Zhejiang, China; ^4^ Human Phenome Institute, Zhangjiang Fudan International Innovation Center, Fudan University, Shanghai, China; ^5^ School of Medicine, Institute of Pharmaceutical Biotechnology, Zhejiang University, Hangzhou, China; ^6^ Joint Institute for Genetics and Genome Medicine, Zhejiang University and University of Toronto, Zhejiang University, Hangzhou, China

**Keywords:** homocysteine, NAFLD, NASH, Cirrhosis, mendelian randomization

## Abstract

**Background:** Since the association of homocysteine and clinical results of observational studies are controversial on non-alcoholic fatty liver related disease, we compute the two-sample Mendelian Randomization (MR) study.

**Objective:** To evaluate whether the plasma level of homocysteine has an effect on the risk of Non-alcoholic fatty liver disease (NAFLD), non-alcoholic steatohepatitis (NASH), and Cirrhosis after its progress, we investigated the causal relationships between plasma homocysteine and the three non-alcoholic fatty liver related diseases mentioned above.

**Design and methods:** Summary estimates were elicited from the inverse-variance weighted (IVW) method through 12 single nucleotide polymorphisms (SNPs) which related to the plasma homocysteine, the SNPs were obtained from a large genome-wide association studies (GWAS) of 44,147 European participants. And the summary statistics for the latest and largest GWAS datasets for NAFLD (307576 in total and 1,578 cases), NASH (309055 in total and 99 cases) and Cirrhosis (306145 in total and 826 cases) were collected from Ristey FinnGen website where the association of genetic variations with blood metabolite levels was conducted using comprehensive metabolite profiling. The study was performed through two-sample MR method.

**Results:** The result indicated that the plasma homocysteine is not significantly associated with NAFLD, and its progression, NASH and Cirrhosis.

**Conclusion:** The evidence in this study is quite deficient to support the causal association of the individual plasma homocysteine with NAFLD, NASH and Cirrhosis, the putative of associations is not exist.

## Introduction

Non-alcoholic fatty liver disease (NAFLD) was firstly used by Ludwig in the 1980s to describe patients diagnosed with fatty degeneration of the liver, which histologically mimics alcoholic hepatitis but without over alcoholic drinking (Ludwig et al., 1980). NAFLD is difficult to diagnose due to the lack of biomarkers (Wong et al., 2015). It has been one of the most commonly faced hepatic disorders all over the world, with an explosive prevalence estimated to be 25% worldwide and 60%–80% in people with risk factors (Younossi et al., 2018). The progression spectrum of NAFLD starts from non-alcoholic fatty liver (NAFL), non-alcoholic steatohepatitis (NASH), and to cirrhosis which is one kind of the end-stage liver disease. And NASH had been the second leading indication for liver transplantation for adults in the United States in 2015 (Wong et al., 2015). Severe comorbidities and sequelae can also be observed, including cardiovascular disease, type 2 diabetes, and NAFLD-related hepatic cancer (Younossi et al., 2019). Previous studies have found highly associated risk factors of NAFLD, while causal evidence is still ambiguous, it is the same for NASH and Cirrhosis. The plasma profile of subjects with NAFLD through untargeted global metabolomic analysis shows higher homocysteine and total cysteine concentration (Kalhan et al., 2011).

Homocysteine (Hcy) is a sulfur-containing amino acid. It can be synthesized from methionine by removing the terminal methyl group, and also be recycled into methionine or converted into cysteine with the aid of certain B-vitamins (Ganguly and Alam, 2015). Elevated serum homocysteine has been observed associated with a series of metabolic disorders, including cerebro-cardiovascular diseases, metabolic syndrome, and venous thromboembolism (VTE) (Catena et al., 2015; Li et al., 2020). Relationship between elevated the homocysteine level and common fatty liver outcomes mentioned above was debated in both cross-sectional and cohort studies (Jansen et al., 2015; Dai et al., 2016; Ventura et al., 2016; Xu et al., 2020). However, these previous studies had been with either small sample sizes or insufficient evidence for causal effect on fatty liver disease, since causal association could not be determined by observational studies. Therefore, firm studies are eagerly needed to explore the causal association between homocysteine and fatty liver progression.

MR is a method of using measured variation in genes of known function to examine the causal effect of a modifiable exposure on disease in observational studies (Davies et al., 2018), which means the MR analysis has more strengths in determining causal relationship in observational studies. It is the use of genetic variants as instrumental variables (IVs) for evaluating causal relationships from GWAS data (Yavorska and Burgess, 2017). MR is a “natural random clinical trial” that can overcome confounders existing in observational studies by randomly dividing people into groups during the gamete formation. Within this project, we aimed to use human genetics to estimate causal effects of homocysteine on the risk of NAFLD and its progression, including NASH and Cirrhosis.

## Methods

### Design of the study

**FIGURE 1:**
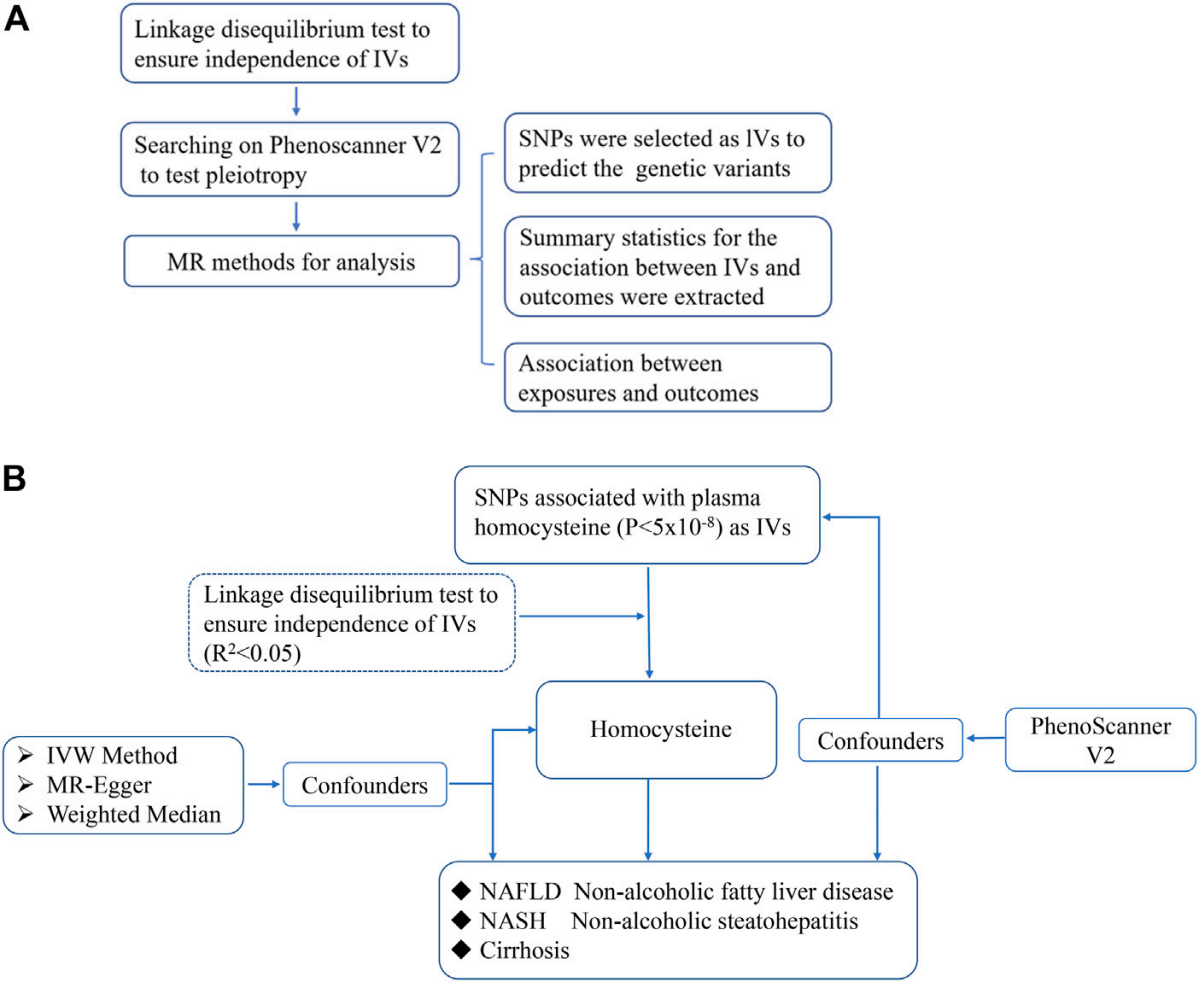
The scientific procedure of Mendelian Randomization analysis. **(A)**. The workflow to compute the relationship of IVs and outcomes in MR; **(B)**. The brief flow chart analysis of serum levels of homocysteine and risk of NAFLD, NASH and Cirrhosis, and NASH and Cirrhosis are more severe form of non-alcoholic fatty liver in progression.

The design of the MR study is shown in flow chart (Figure 1A), which is based on three assumptions, and these assumptions must be plausibly assessed (Davies et al., 2018): the relevance assumption, independence and exclusion restriction assumptions. To be specific, the selected SNPs are the IVs that can predict the genetic variants, and also, the linkage disequilibrium and the pleiotropy of IVs are tested (Figure 1B). To assess the associations between homocysteine and risks of NAFLD, NASH and Cirrhosis. SNPs of homocysteine were identified at genome-wide significance (*p* < 5 × 10^–8^) from the hitherto largest GWAS meta-analysis, with up to 44,147 individuals of European ancestry (van Meurs et al., 2013).

### Exposure SNP selection

Multiple genetic variants associated with plasma homocysteine were selected (r2 < 0.8, *p* < 5.0 × 10^–8^) from the largest genome-wide association studies (GWAS). The meta-analysis included 10 cohorts collected from different studies. Plasma homocysteine levels were measured in each cohort by different chromatography and spectrometry techniques such as isotope-dilution liquid chromatography–tandem mass spectrometry, gas chromatography–coupled mass spectrometry, high-performance liquid chromatography (HPLC), or enzymatic, immune, or chemiluminescence (Ganguly and Alam, 2015). Each cohort of the participants was genotyped and the exclusion criteria of SNPs were set (Ganguly and Alam, 2015). The age composition of the participants in each cohort is between 17 and 79, and the sex composition is roughly balanced with women taking part in about 45–58%. Here, 18 SNPs were chosen as IVs, the independent contribution of selected SNPs was tested in Linkage disequilibrium (LD)-link website (https://ldlink.nci.nih.gov/, population: CEU), which was a suite of web-based applications designed to examine LD in population groups, and we use LD < 0.05 as a threshold to ensure the SNPs we selected were independent. Three of the 18 SNPs were excluded (rs12134663, rs957140, rs12921383) (Supplementary Tables S1–6), and potential confounding factors were examined by PhenoScanner (Bowden et al., 2016; Lee et al., 2016) to exclude instrument variants with significant effects on each outcome, while none of the SNPs were eliminated. And another three SNPs (rs7422339, rs2851391, rs12780845) were lack of information in the GWAS. The detail information of the 12 SNPs we chose is shown in the table below (Table 1).

**TABLE 1:** Related traits of homocysteine-associated SNPs.

SNPs	Nearest	Chr	EA	EAF	Association with hcy			Association with outcomes					
								NAFLD		NASH		Cirrhosis	
	gene				beta	se	*p*-value	beta	se	beta	se	beta	se
rs1801133	MTHFR	1	A	0.34	0.1583	0.007	4.34E-104	0.063	0.042	−0.037	0.168	−0.142	0.058
rs2275565	MTR	1	T	0.21	-0.0542	0.009	1.96E-10	0.044	0.044	−0.020	0.173	−0.020	0.060
rs9369898	MUT	6	A	0.62	0.0449	0.007	2.17E-10	−0.012	0.037	0.297	0.146	0.052	0.050
rs7130284	NOX4	11	T	0.07	-0.1242	0.013	1.88E-20	−0.008	0.053	−0.244	0.210	0.029	0.072
rs154657	DPEP1	16	A	0.45	0.0963	0.007	1.74E-43	0.043	0.037	0.144	0.147	0.054	0.051
rs4660306	MMACHC	1	T	0.33	0.0435	0.007	2.33E-09	0.000	0.038	−0.036	0.150	0.102	0.052
rs548987	SLC17A3	6	C	0.13	0.0597	0.01	1.12E-08	0.014	0.063	−0.216	0.251	-0.114	0.087
rs42648	GTPB10	7	A	0.40	-0.0395	0.007	1.97E-08	-0.013	0.037	-0.069	0.147	0.000	0.051
rs1801222	CUBN	10	A	0.34	0.0453	0.007	8.43E-10	0.049	0.038	−0.057	0.153	-0.030	0.053
rs2251468	HNF1A	12	A	0.65	-0.0512	0.007	1.28E-12	-0.047	0.037	0.252	0.146	0.026	0.050
rs838133	FUT2	19	A	0.45	0.0422	0.007	7.48E-09	0.013	0.037	0.099	0.148	0.025	0.051
rs12780845	CUBN	10	A	0.65	0.0529	0.009	7.80E-10	-0.081	0.041	0.236	0.165	-0.021	0.057

Chr., chromosome; EA, effect allele; EAF, effect allele frequency.

### Genetic association with outcomes

The outcomes include NAFLD, NASH and Cirrhosis. Summary data for NAFLD was extracted from the largest GWAS meta-analysis including 1,578 cases and 307576 controls of European ancestry (https://r7.risteys.finngen.fi/phenocode/NAFLD), statistics for NASH including 99 cases and 309055 controls (https://r7.risteys.finngen.fi/phenocode/NASH), and statistics for Cirrhosis including 826 cases and 306145 controls (https://r7.risteys.finngen.fi/phenocode/CHIRHEP_NAS). The analyses for this study were all based on publicly available summary statistics. The participants have provided written consent, and all the studies contributing data to our MR analysis were approved by the relevant ethical review boards.

### Statistical analysis and sensitivity analysis

The primary MR analysis of the causal association between homocysteine and NAFLD, NASH and Cirrhosis progression outcomes was performed, and each IV’s combined causal effects were estimated by IVW approach. The IVW approach is that the combined effect was evaluated by calculating the Wald ratio of each SNP, and then using the corresponding inverse variance as weights for meta-analysis (Lee et al., 2016). In sensitivity analysis, we use a weighted median (WM) approach which calculates the median value of the IVs’ estimates (Bowden et al., 2016) and MR-Presso analysis (Verbanck et al., 2018). In addition, the pleiotropic effects were tested through the MR-Egger approach, which investigates whether the intercept of the association between plasma homocysteine and NAFLD progression differs from zero (Bowden et al., 2015).

All statistical analyses in the research were conducted by R version 4.0.2 and R package named “MendelianRandomization”.

## Result

### Mendelian randomization analysis on the impact of NAFLD, NASH and cirrhosis on the homocysteine

We performed IVW analyses to explore the causal associations between homocysteine concentrations and NAFLD, NASH, as well as Cirrhosis, and the results showed the causal effects of genetic variants of homocysteine concentration in plasma and all the outcomes (Supplementary Tables S7). The putative of associations was not exist.

**FIGURE 2:**
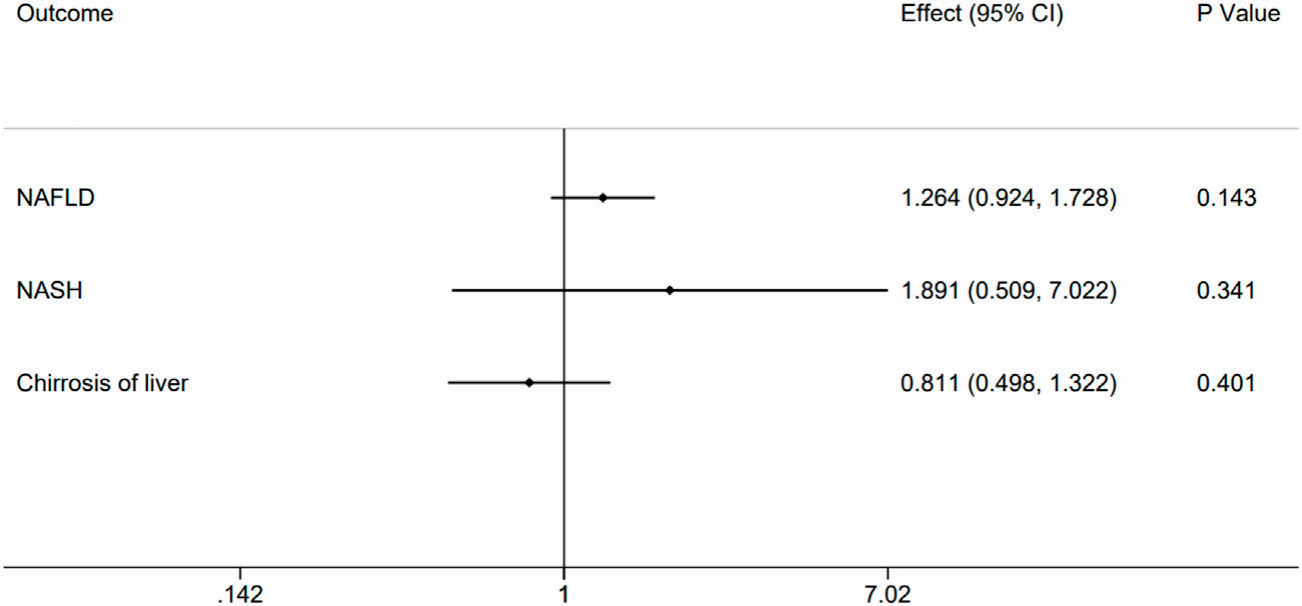
Causal impact of genetically predicted non-alcoholic fatty related liver diseases, NAFLD, NASH and Cirrhosis using 12 genome-wide significant SNPs on the plasma homocysteine. Forest plot depicting the effect of genetically predicted NAFLD, NASH and Cirrhosis on plasma homocysteine (*n* = 12) using inverse-variance weighted Mendelian randomization.

Specifically, the outcomes of homocysteine level in plasma showed no evidence to support causal association (Figure 2) on NAFLD (effect = 1.264, 95% CI:0.924–1.728, *p* = 0.143), NASH (effect = 1.891, 95% CI:0.509–7.022, *p* = 0.341), and Cirrhosis (effect = 0.811, 95% CI:0.498–1.322, *p* = 0.401). There was no relationship between homocysteine levels and overall NAFLD, NASH and Cirrhosis. The effect of every SNP was demonstrated in the Supplementary Figure S1.

### Sensitivity analysis

**FIGURE 3:**
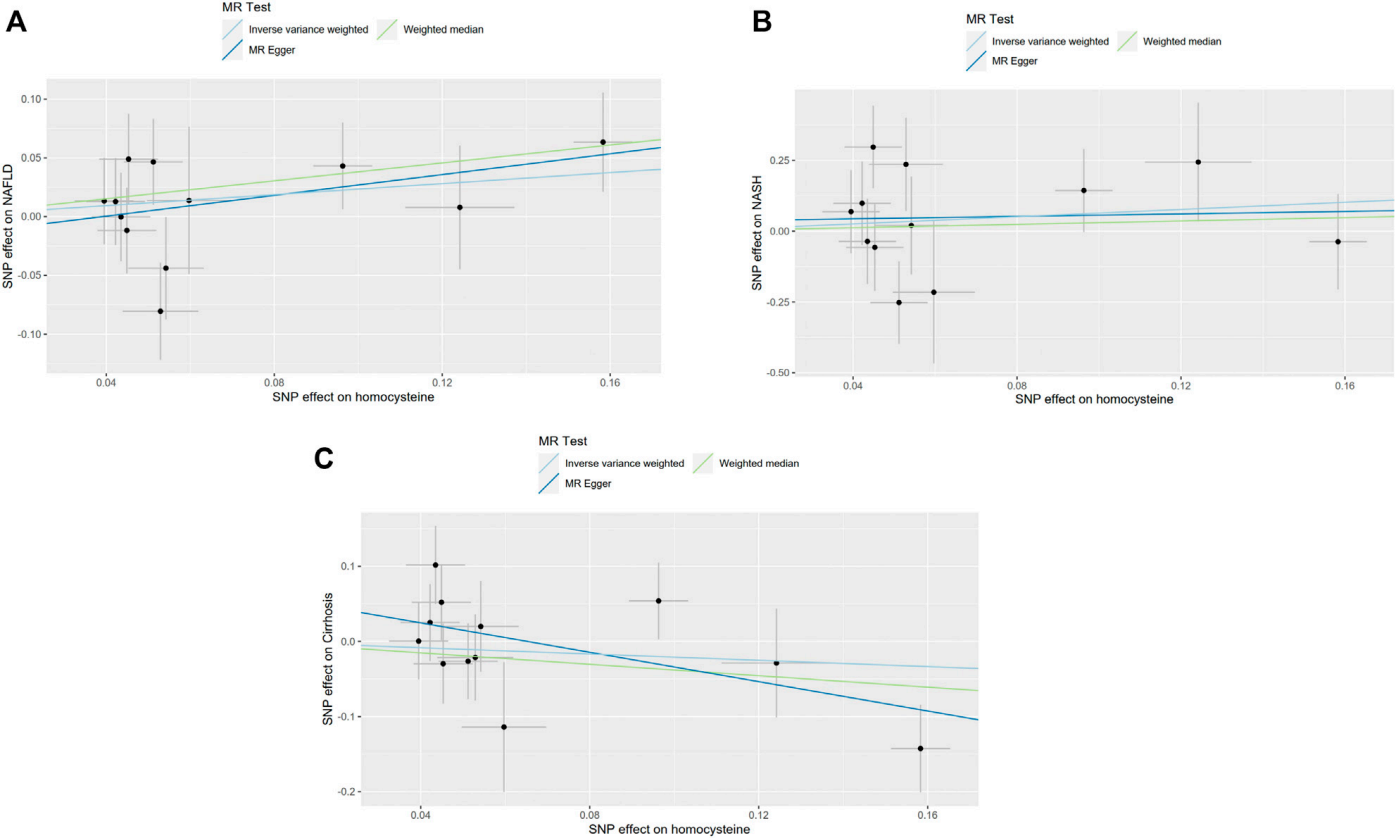
Single MR estimates from each of the genetic variants using IVW, WM and MR-Egger methods. The black scatter plots indicate single causal estimates from each of the genetic variants associated with plasma homocysteine level on the *x*-axis and the outcomes, NAFLD, NASH and Cirrhosis on the *y*-axis. The continuous line represents the causal effect of plasma homocysteine on NAFLD, NASH, and Cirrhosis, separately. **(A)**. Non-alcoholic fatty liver disease (NAFLD); **(B)**. Non-alcoholic steatohepatitis (NASH); **(C)**. Cirrhosis.

In order to confirm the robustness of the causal association between homocysteine and NAFLD, NASH and also Cirrhosis, the sensitivity analysis was processed through WM, MR-Egger and MR-Presso analysis (Verbanck et al., 2018). The results of WM, MR-Egger and MR-Presso methods were quite close to the results calculated by IVW analyses (Supplementary Table S8). What’s more, the intercept values of the MR-Egger analysis did not significantly differ from zero. The sensitivity analysis indicated that in the pleiotropy aspect, our results were not biased. Single MR estimates from each of the genetic variants using IVW, WM and MR-Egger method were also shown below (Figure 3).

## Discussion

NAFLD is one of the most common chronic liver diseases worldwide (Masuoka and Chalasani, 2013), especially in Western countries, it featured a broad spectrum of conditions (Eslam et al., 2018), and what’s more, growing evidence shows that NAFLD is a multisystem disease, involving other organs and regulatory pathways. The factors related to the progression of liver disease in NAFLD and the factors linking NAFLD with other extra-hepatic chronic diseases (Byrne and Targher, 2015) are necessary to be focused and clarified. However, diagnostics on a case with NAFLD loads lots of burdens, especially in clinical trials. Multiple techniques are applied to assistant diagnosis such as radiological imaging (Lee and Park, 2014), ultrasonography (Hernaez et al., 2011), or magnetic resonance techniques (Szczepaniak et al., 2005; Reeder et al., 2011), and there are histological characterization of the liver by a designated scoring system (Kleiner et al., 2005; Sumida et al., 2014). Despite the convenience and accuracy, most of the non-invasive methods are more suitable for measuring liver fat, not definitely to diagnose a new case in the early stage of NAFLD. And the progressive form of NAFLD, NASH and Cirrhosis, the diagnosis necessitates invasive liver biopsy (Younossi et al., 2018).

Various factors are involved in the development and progress of NAFLD. As the largest solid organ, liver is constantly exposed to circulating nutrients and endotoxins derived from the gut microbiota (Wang et al., 2021), and it suffers burdens from all over the body, obviously, various metabolism nutrition, endotoxins and waste, are also processed by liver. The impairment of liver often starts from oxidation and inflammation. Increased oxidative stress plays a role in the pathogenesis of NAFLD (Yang et al., 2019; Chen et al., 2020), and the cellular antioxidant was abnormal such as glutathione (GSH) (Rom et al., 2020), meanwhile, the peroxisome proliferator-activated receptors (PPARs) are also involved (Francque et al., 2021).

Hepatic cystathionine-β-synthase (CBS) and cystathionine-γ-lyase (CSE) system (CBS/CSE system) is an emerging therapeutic target in NAFLD for it is the system which regulates homocysteine and cysteine metabolism and it is a key role in endogenous hydrogen sulfide (H2S) biosynthesis (Sarna et al., 2015), which means in some way, the level of homocysteine and its metabolism could be related to liver damage like NAFLD, NASH and Cirrhosis. The high-performance liquid chromatography (HPLC) with fluorescence detection was performed to analyze plasma homocysteine as part of plasma thiols among 63 NAFLD patients, and the result shows that the homocysteine plasma level increases, and as in children, the homocysteine and cysteine, and the presence of fibrosis demonstrated a positive correlation (Pastore et al., 2014). More than that, NAFLD patients have problems with absorption of Vitamin B12, and homocysteine accumulates in plasma when vitamin B12 is in short. The original study of relationship among vitamin B12 levels, homocysteine levels and NAFLD shows that vitamin B12 levels and NAFLD have no obvious association, but patients with NAFLD have significantly higher homocysteine level as a potential marker for liver damage (Costa et al., 2021). However, there are also conclusions from observation studies in dispute (Polyzos et al., 2012; Polyzos et al., 2020; Xu et al., 2020).

For reasons not limited to those we have demonstrated above, important genetic and epigenetic modifiers of NAFLD progression provide the potential to design novel therapeutics and the clinical implementation of diagnostic/prognostic biomarkers (Byrne and Targher, 2015). To understand the genetic basis of NAFLD is a new approach to improve the diagnosis and explore the etiology. In advantage of multiple genome-wide association and large candidate gene studies, we compute MR for the association between homocysteine and NAFLD, NASH and Cirrhosis, to test if homocysteine is the causation of these diseases in progression, the results show no significant causal effect association among them, therefore, homocysteine is not the causal role in the etiology of NAFLD, NASH and Cirrhosis. The results demonstrate that the observational association between homocysteine and NAFLD, and its progression is probably caused by confounders or reverse causation.

Our study still has some limitations. On one hand, patients and controls for the analysis are all from Europe whose genetic characteristic may differ from other populations, which make the generalizability discount to our study, and most ongoing prospective studies are probably too limited to find disease markers (Krawczak et al., 2006), therefore, evaluating whether the plasma level of homocysteine is the maker of NAFLD and its progression, we had better broaden the resources of the patients and controls. On the other hand, with the incidence increasing year by year (Allen et al., 2018), and NAFLD is quite a complex disease which is difficult to diagnose (Friedman et al., 2018), some of the approaches to diagnose NAFLD was not confirmed by liver imaging or histology (Machado and Cortez-Pinto, 2014; St. Pierre et al., 2016), consequently, these mean that there are probably chances to misclassification of cases and controls (Barritt et al., 2021) or take a more severe form of the disease as an average patient with NAFLD. Last but not the least, the IVs of homocysteine need to be expanded to meet the multifactorial and complex disease like NAFLD, NASH and Cirrhosis. Though this study did not show a causal association, considering that the etiology of NASH is complex and lacks of animal models, MR method still can serve as a good alternative to experimental research.

In summary, the results of our Mendelian Randomization study do not suggest a causal role of plasma homocysteine levels in NAFLD and its progression, NASH and Cirrhosis as well.

## Data Availability

The original contributions presented in the study are included in the article/Supplementary Material, further inquiries can be directed to the corresponding author.

## References

[B1] Allen A. M. Therneau T. M. Larson J. J. Coward A. Somers V. K. Kamath P. S. (2018). Nonalcoholic fatty liver disease incidence and impact on metabolic burden and death: A 20 year-community study. Hepatology 67 (5), 1726–1736. 10.1002/hep.29546 28941364 PMC5866219

[B2] Barritt A. S. Watkins S. Gitlin N. Klein S. Lok A. S. Loomba R. (2021). Patient determinants for histologic diagnosis of NAFLD in the real world: A TARGET-NASH study. Hepatol. Commun. 5 (6), 938–946. 10.1002/hep4.1689 34141981 PMC8183178

[B3] Bowden J. Davey Smith G. Burgess S. (2015). Mendelian randomization with invalid instruments: Effect estimation and bias detection through egger regression. Int. J. Epidemiol. 44 (2), 512–525. 10.1093/ije/dyv080 26050253 PMC4469799

[B4] Bowden J. Davey Smith G. Haycock P. C. Burgess S. (2016). Consistent estimation in mendelian randomization with some invalid instruments using a weighted median estimator. Genet. Epidemiol. 40 (4), 304–314. 10.1002/gepi.21965 27061298 PMC4849733

[B5] Byrne C. D. Targher G. (2015). Nafld: A multisystem disease. J. Hepatol. 62, S47–S64. 10.1016/j.jhep.2014.12.012 25920090

[B6] Catena C. Colussi G. Nait F. Capobianco F. Sechi L. A. (2015). Elevated homocysteine levels are associated with the metabolic syndrome and cardiovascular events in hypertensive patients. Am. J. Hypertens. 28 (7), 943–950. 10.1093/ajh/hpu248 25498997

[B7] Chen Z. Tian R. She Z. Cai J. Li H. (2020). Role of oxidative stress in the pathogenesis of nonalcoholic fatty liver disease. Free Radic. Biol. Med. 152, 116–141. 10.1016/j.freeradbiomed.2020.02.025 32156524

[B8] Costa D. S. Guahnon M. P. Seganfredo F. B. Pinto L. P. Tovo C. V. Fernandes S. A. (2021). Vitamin B12 and homocysteine levels in patients with NAFLDa systemtic review and metanalysis. Arq. Gastroenterol. 58 (2), 234–239. 10.1590/s0004-2803.202100000-42 34287533

[B9] Dai H. Wang W. Tang X. Chen R. Chen Z. Lu Y. (2016). Association between homocysteine and non-alcoholic fatty liver disease in Chinese adults: A cross-sectional study. Nutr. J. 15 (1), 102. 10.1186/s12937-016-0221-6 27955646 PMC5153832

[B10] Davies N. M. Holmes M. V. Davey Smith G. (2018). Reading mendelian randomisation studies: A guide, glossary, and checklist for clinicians. Gloss. Checkl. Clin. 362, k601. 10.1136/bmj.k601 PMC6041728 30002074

[B11] Eslam M. Valenti L. Romeo S. (2018). Genetics and epigenetics of NAFLD and NASH: Clinical impact. J. Hepatol. 68 (2), 268–279. 10.1016/j.jhep.2017.09.003 29122391

[B12] Francque S. Szabo G. Abdelmalek M. F. Byrne C. D. Cusi K. Dufour J. F. (2021). Nonalcoholic steatohepatitis: The role of peroxisome proliferator-activated receptors. Nat. Rev. Gastroenterol. Hepatol. 18 (1), 24–39. 10.1038/s41575-020-00366-5 33093663

[B13] Friedman S. L. Neuschwander-Tetri B. A. Rinella M. Sanyal A. J. (2018). Mechanisms of NAFLD development and therapeutic strategies. Nat. Med. 24 (7), 908–922. 10.1038/s41591-018-0104-9 29967350 PMC6553468

[B14] Ganguly P. Alam S. F. (2015). Role of homocysteine in the development of cardiovascular disease. Nutr. J. 14, 6. 10.1186/1475-2891-14-6 25577237 PMC4326479

[B15] Hernaez R. Lazo M. Bonekamp S. Kamel I. Brancati F. L. Guallar E. (2011). Diagnostic accuracy and reliability of ultrasonography for the detection of fatty liver: A meta-analysis. Hepatology 54 (3), 1082–1090. 10.1002/hep.24452 21618575 PMC4197002

[B16] Jansen E. Beekhof P. Viezeliene D. Muzakova V. Skalicky J. (2015). Long-term stability of cancer biomarkers in human serum: Biomarkers of oxidative stress and redox status, homocysteine, CRP and the enzymes ALT and GGT. Biomark. Med. 9 (5), 425–432. 10.2217/bmm.15.14 25985173

[B17] Kalhan S. C. Guo L. Edmison J. Dasarathy S. McCullough A. J. Hanson R. W. (2011). Plasma metabolomic profile in nonalcoholic fatty liver disease. Metabolism. 60 (3), 404–413. 10.1016/j.metabol.2010.03.006 20423748 PMC2950914

[B18] Kleiner D. E. Brunt E. M. Van Natta M. Behling C. Contos M. J. Cummings O. W. (2005). Design and validation of a histological scoring system for nonalcoholic fatty liver disease. Hepatology 41 (6), 1313–1321. 10.1002/hep.20701 15915461

[B19] Krawczak M. Nikolaus S. von Eberstein H. Croucher P. J. P. El Mokhtari N. E. Schreiber S. (2006). PopGen: Population-Based recruitment of patients and controls for the analysis of complex genotype-phenotype relationships. Community Genet. 9 (1), 55–61. 10.1159/000090694 16490960

[B20] Lee C. H. Cook S. Lee J. S. Han B. (2016). Comparison of two meta-analysis methods: Inverse-Variance-Weighted average and weighted sum of Z-scores. Genomics Inf. 14 (4), 173–180. 10.5808/gi.2016.14.4.173 PMC5287121 28154508

[B21] Lee S. S. Park S. H. (2014). Radiologic evaluation of nonalcoholic fatty liver disease. World J. Gastroenterol. 20 (23), 7392–7402. 10.3748/wjg.v20.i23.7392 24966609 PMC4064084

[B22] Li S. Sun L. Qi L. Jia Y. Cui Z. Wang Z. (2020). Effect of high homocysteine level on the severity of coronary heart disease and prognosis after stent implantation. J. Cardiovasc. Pharmacol. 76 (1), 101–105. 10.1097/fjc.0000000000000829 32304562

[B23] Ludwig J. Viggiano T. R. McGill D. B. Oh B. J. (1980). Nonalcoholic steatohepatitis: Mayo Clinic experiences with a hitherto unnamed disease. Mayo Clin. Proc. 55 (7), 434–438. 7382552

[B24] Machado M. V. Cortez-Pinto H. (2014). Non-alcoholic fatty liver disease: What the clinician needs to know. World J. Gastroenterol. 20 (36), 12956–12980. 10.3748/wjg.v20.i36.12956 25278691 PMC4177476

[B25] Masuoka H. C. Chalasani N. (2013). Nonalcoholic fatty liver disease: An emerging threat to obese and diabetic individuals. Ann. N. Y. Acad. Sci. 1281 (1), 106–122. 10.1111/nyas.12016 23363012 PMC3646408

[B26] Pastore A. Alisi A. di Giovamberardino G. Crudele A. Ceccarelli S. Panera N. (2014). Plasma levels of homocysteine and cysteine increased in pediatric NAFLD and strongly correlated with severity of liver damage. Int. J. Mol. Sci. 15 (11), 21202–21214. 10.3390/ijms151121202 25407526 PMC4264220

[B27] Polyzos S. A. Kountouras J. Patsiaoura K. Katsiki E. Zafeiriadou E. Deretzi G. (2012). Serum homocysteine levels in patients with nonalcoholic fatty liver disease. Ann. Hepatol. 11 (1), 68–76. 10.1016/s1665-2681(19)31488-7 22166563

[B28] Polyzos S. A. Papaefthymiou A. Doulberis M. Katsinelos P. Kountouras J. (2020). Homocysteine in nonalcoholic steatohepatitis: Seemingly a paradox revisited. J. Gastrointestin. Liver Dis. 29 (2), 270–271. 10.15403/jgld-1667 32530998

[B29] Reeder S. B. Cruite I. Hamilton G. Sirlin C. B. (2011). Quantitative assessment of liver fat with magnetic resonance imaging and spectroscopy. J. Magn. Reson. Imaging 34 (4), 729–749. 10.1002/jmri.22775 22025886 PMC3177109

[B30] Rom O. Liu Y. Liu Z. Zhao Y. Wu J. Ghrayeb A. (2020). Glycine-based treatment ameliorates NAFLD by modulating fatty acid oxidation, glutathione synthesis, and the gut microbiome. Sci. Transl. Med. 12 (572), eaaz2841. 10.1126/scitranslmed.aaz2841 33268508 PMC7982985

[B31] Sarna L. K. Siow Y. L. O K. (2015). The CBS/CSE system: A potential therapeutic target in. Can. J. Physiol. Pharmacol. 93 (1), 1–11. 10.1139/cjpp-2014-0394 25493326

[B32] St. Pierre T. G. House M. J. Bangma S. J. Pang W. Bathgate A. Gan E. K. (2016). Stereological analysis of liver biopsy histology sections as a reference standard for validating non-invasive liver fat fraction measurements by MRI. PLoS One 11 (8), e0160789. 10.1371/journal.pone.0160789 27501242 PMC4976876

[B33] Sumida Y. Nakajima A. Itoh Y. (2014). Limitations of liver biopsy and non-invasive diagnostic tests for the diagnosis of nonalcoholic fatty liver disease/nonalcoholic steatohepatitis. World J. Gastroenterol. 20 (2), 475–485. 10.3748/wjg.v20.i2.475 24574716 PMC3923022

[B34] Szczepaniak L. S. Nurenberg P. Leonard D. Browning J. D. Reingold J. S. Grundy S. (2005). Magnetic resonance spectroscopy to measure hepatic triglyceride content: Prevalence of hepatic steatosis in the general population. Am. J. Physiol. Endocrinol. Metab. 288 (2), E462–E468. 10.1152/ajpendo.00064.2004 15339742

[B35] van Meurs J. B. Pare G. Schwartz S. M. Hazra A. Tanaka T. Vermeulen S. H. (2013). Common genetic loci influencing plasma homocysteine concentrations and their effect on risk of coronary artery disease. Am. J. Clin. Nutr. 98 (3), 668–676. 10.3945/ajcn.112.044545 23824729 PMC4321227

[B36] Ventura P. Venturelli G. Marcacci M. Fiorini M. Marchini S. Cuoghi C. (2016). Hyperhomocysteinemia and MTHFR C677T polymorphism in patients with portal vein thrombosis complicating liver cirrhosis. Thromb. Res. 141, 189–195. 10.1016/j.thromres.2016.03.024 27065203

[B37] Verbanck M. Chen C. Y. Neale B. Do R. (2018). Detection of widespread horizontal pleiotropy in causal relationships inferred from Mendelian randomization between complex traits and diseases. Nat. Genet. 50 (5), 693–698. 10.1038/s41588-018-0099-7 29686387 PMC6083837

[B38] Wang R. Tang R. Li B. Ma X. Schnabl B. Tilg H. (2021). Gut microbiome, liver immunology, and liver diseases. Cell. Mol. Immunol. 18 (1), 4–17. 10.1038/s41423-020-00592-6 33318628 PMC7852541

[B39] Wong R. J. Aguilar M. Cheung R. Perumpail R. B. Harrison S. A. Younossi Z. M. (2015). Nonalcoholic steatohepatitis is the second leading etiology of liver disease among adults awaiting liver transplantation in the United States. Gastroenterology 148 (3), 547–555. 10.1053/j.gastro.2014.11.039 25461851

[B40] Xu Y. Guan Y. Yang X. Xia Z. Wu J. (2020). Association of serum homocysteine levels with histological severity of NAFLD. J. Gastrointestin. Liver Dis. 29 (1), 51–58. 10.15403/jgld-529 32176759

[B41] Yang J. Fernández-Galilea M. Martínez-Fernández L. González-Muniesa P. Pérez-Chávez A. Martínez J. A. (2019). Oxidative stress and non-alcoholic fatty liver disease: Effects of omega-3 fatty acid supplementation. Nutrients 11 (4), 872. 10.3390/nu11040872 31003450 PMC6521137

[B42] Yavorska O. O. Burgess S. (2017). MendelianRandomization: an R package for performing Mendelian randomization analyses using summarized data. Int. J. Epidemiol. 46 (6), 1734–1739. 10.1093/ije/dyx034 28398548 PMC5510723

[B43] Younossi Z. Anstee Q. M. Marietti M. Hardy T. Henry L. Eslam M. (2018). Global burden of NAFLD and NASH: Trends, predictions, risk factors and prevention. Nat. Rev. Gastroenterol. Hepatol. 15 (1), 11–20. 10.1038/nrgastro.2017.109 28930295

[B44] Younossi Z. Tacke F. Arrese M. Chander Sharma B. Mostafa I. Bugianesi E. (2019). Global perspectives on nonalcoholic fatty liver disease and nonalcoholic steatohepatitis. Hepatology 69 (6), 2672–2682. 10.1002/hep.30251 30179269

