# Supplementary figures and images for: Effects of homocysteine on nonalcoholic fatty liver related disease: A mendelian randomization study
Source: Front Mol Biosci. 2022 Dec 6;9:1083855. doi: 10.3389/fmolb.2022.1083855 (PMC9763576; doi:10.3389/fmolb.2022.1083855)

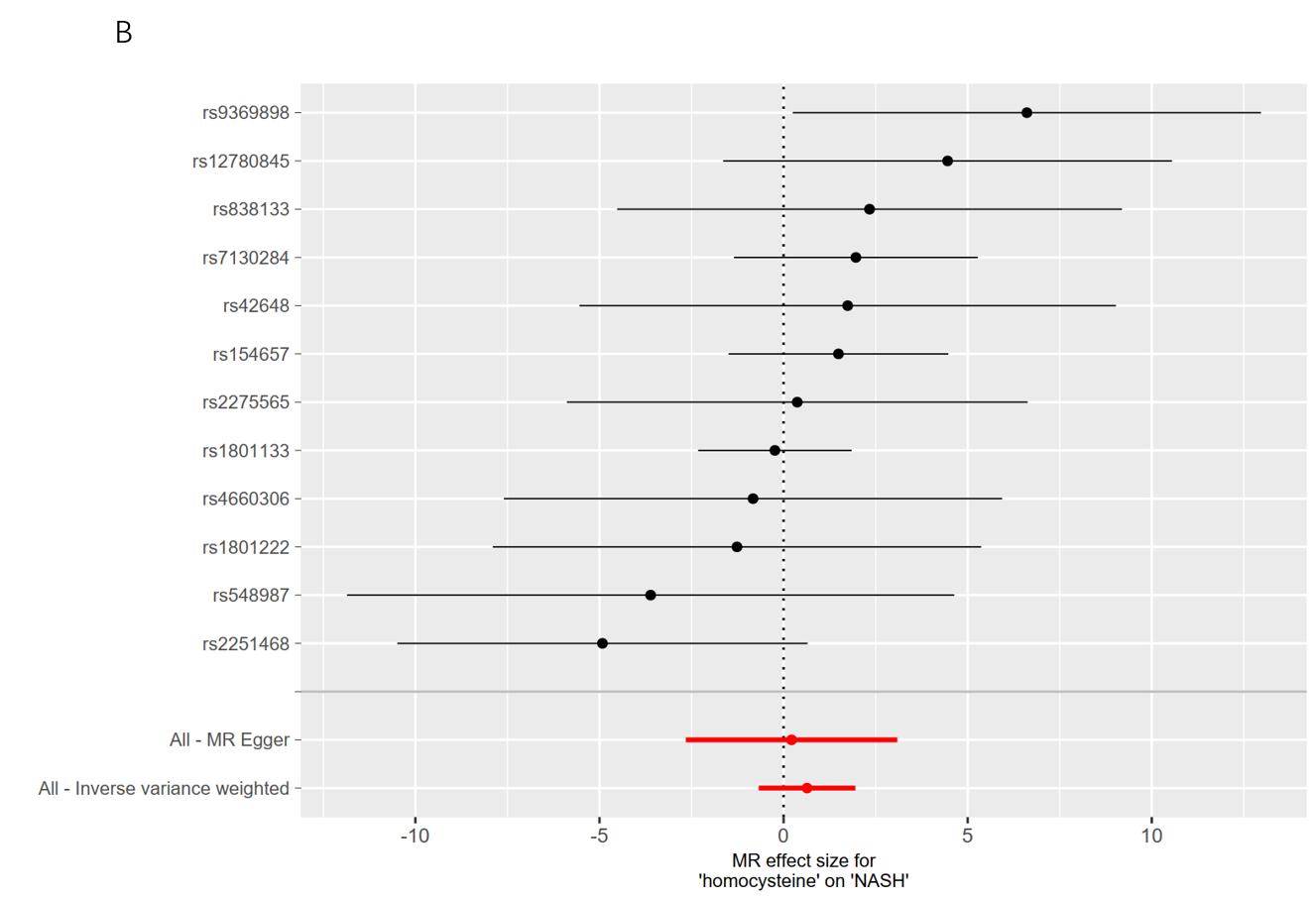

Supplement: Supplementary file 1 [file Image2.PNG]

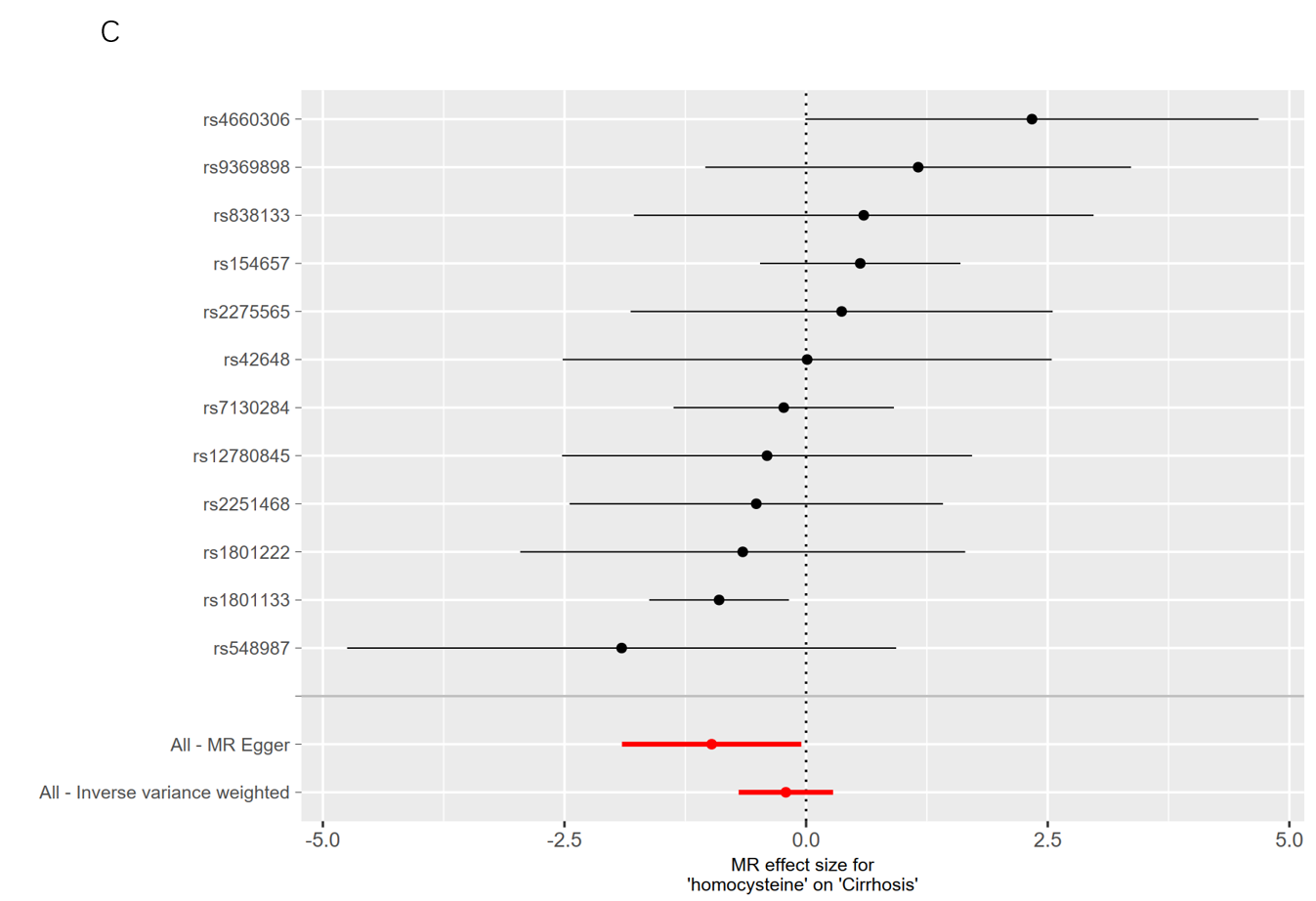

Supplement: Supplementary file 2 [file Image3.PNG]

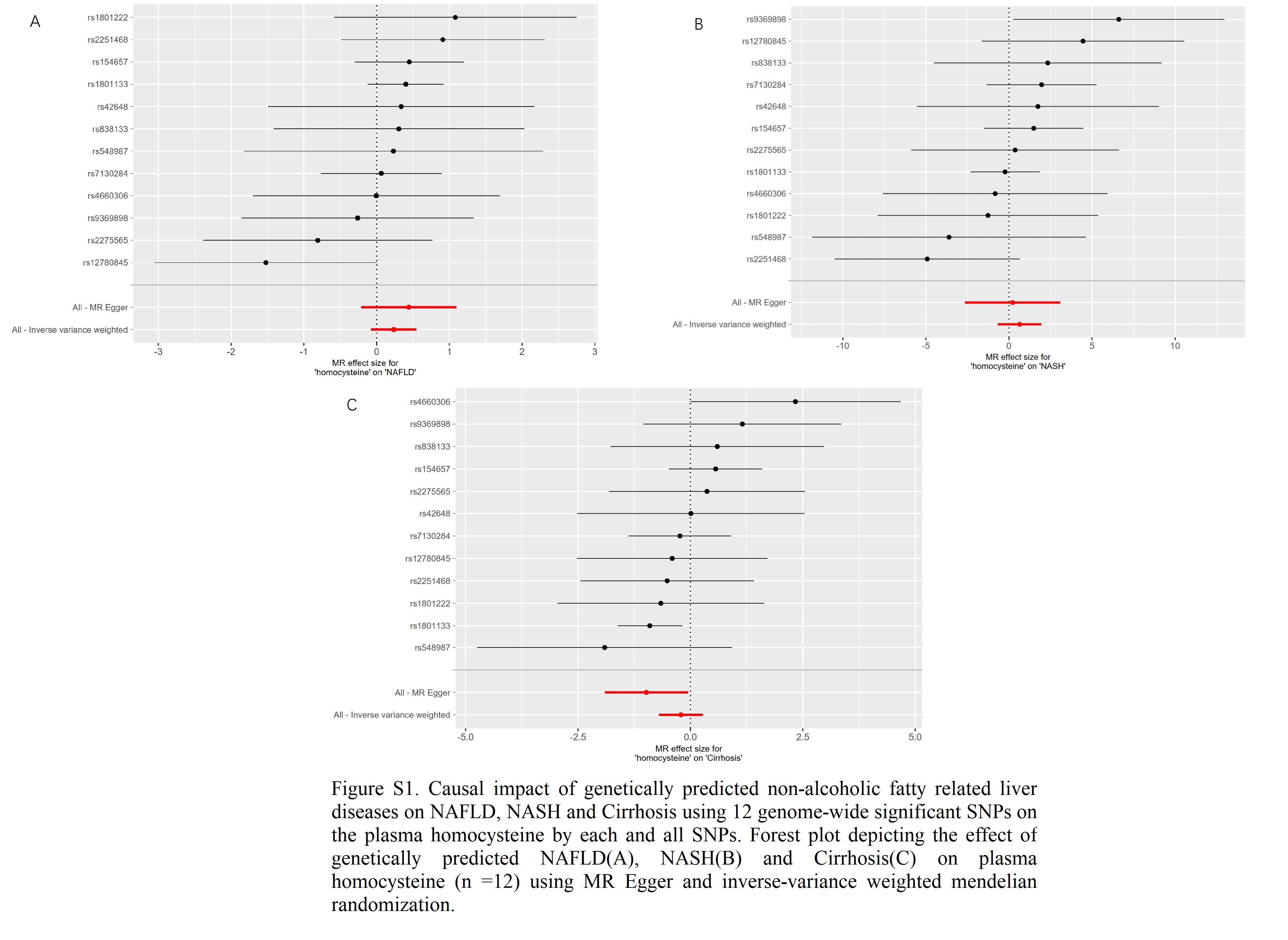

Supplement: Supplementary file 3 [file Image1.jpg]
