# Supplementary material for: Effects of homocysteine on nonalcoholic fatty liver related disease: A mendelian randomization study
Source: Front Mol Biosci. 2022 Dec 6;9:1083855. doi: 10.3389/fmolb.2022.1083855 (PMC9763576; doi:10.3389/fmolb.2022.1083855)
Supplement: Supplementary file 4 [file DataSheet1.docx]

**Table S1 Calculation of linkage disequilibrium of the selected SNP on chromosome 1**

| RS_number | rs12134663 | rs1801133 | rs4660306 | rs2275565 |
| --- | --- | --- | --- | --- |
| rs12134663 | 1 | 0.094 | 0.012 | 0.002 |
| rs1801133 | 0.094 | 1 | 0.015 | 0.011 |
| rs4660306 | 0.012 | 0.015 | 1 | 0.001 |
| rs2275565 | 0.002 | 0.011 | 0.001 | 1 |

The estimates of linkage disequilibrium are reported as r^2^ values in the table.

Population = (CEU) Utah Residents from North and West Europe; r^2^ < 0.005.

**Table S2 Calculation of linkage disequilibrium of the selected SNP on chromosome 10**

| SNP | rs1801222 | rs12780845 |
| --- | --- | --- |
| rs1801222 | 1.0 | 0.007 |
| rs12780845 | 0.007 | 1.0 |

The estimates of linkage disequilibrium are reported as r^2^ values in the table.

Population = (CEU) Utah Residents from North and West Europe; r^2^ < 0.005.

**Table S3 Calculation of linkage disequilibrium of the selected SNP on chromosome 11**

| SNP | rs7130284 | rs957140 |
| --- | --- | --- |
| rs7130284 | 1.0 | 0.05 |
| rs957140 | 0.05 | 1.0 |

The estimates of linkage disequilibrium are reported as r^2^ values in the table.

Population = (CEU) Utah Residents from North and West Europe; r^2^ < 0.005.

**Table S4 Calculation of linkage disequilibrium of the selected SNP on chromosome 16**

| SNP | rs154657 | rs12921383 |
| --- | --- | --- |
| rs154657 | 1.0 | 0.111 |
| rs12921383 | 0.111 | 1.0 |

The estimates of linkage disequilibrium are reported as r^2^ values in the table.

Population = (CEU) Utah Residents from North and West Europe; r^2^ < 0.005.

**Table S5 Calculation of linkage disequilibrium of the selected SNP on chromosome 6**

| SNP | rs548987 | rs9369898 |
| --- | --- | --- |
| rs548987 | 1.0 | 0.003 |
| rs9369898 | 0.003 | 1.0 |

The estimates of linkage disequilibrium are reported as r^2^ values in the table.

Population = (CEU) Utah Residents from North and West Europe; r^2^ < 0.005.

**Table S6 Calculation of linkage disequilibrium of the selected SNP on chromosome 21**

| SNP | rs234709 | rs2851391 |
| --- | --- | --- |
| rs234709 | 1.0 | 0.006 |
| rs2851391 | 0.006 | 1.0 |

The estimates of linkage disequilibrium are reported as r^2^ values in the table.

Population = (CEU) Utah Residents from North and West Europe; r^2^ < 0.005.

**Table S7 The causal effects the genetic variants of homocysteine concentration in plasma and all the outcomes by** **Inverse-variance weighted method.**

| Outcome | Cases/controls | Estimate | Std Error | 95% CI | P-value |
| --- | --- | --- | --- | --- | --- |
| NAFLD | 1578/307576 | 0.234 | 0.160 | -0.079, 0.547 | 0.143 |
| NASH | 99/309055 | 0.637 | 0.670 | -0.675, 1.949 | 0.341 |
| Cirrhosis | 826/306145 | -0.209 | 0.249 | -0.698, 0.279 | 0.401 |

Non-alcoholic fatty liver disease (NAFLD), Non-alcoholic steatohepatitis (NASH).

**Table S8 Weighted median, MR-Egger and MR-Presso analysis for genetic associations between exposures and outcomes**

| Method | Weighted median | MR-Egger | | MR-Presso |
| --- | --- | --- | --- | --- |
|  |  | Estimate | Intercept |  |
| NAFLD | | | |  |
| Estimate (95% CI) | 0.208 (-0.027, 0.790) | 0.335 (-0.215, 1.099) |  |  |
| P value | 0.068 | 0.187 | 0.481 | 0.547 |
| NASH | | | |  |
| Estimate (95% CI) | 0.842 (-1.342, 1.959) | 1.466 (-2.656, 3.090) |  |  |
| P value | 0.714 | 0.882 | 0.744 | 0.355 |
| Cirrhosis | | | |  |
| Estimate (95% CI) | 0.325 (-1.018, 0.257) | 0.473 (-1.905, -0.049) |  |  |
| P value | 0.242 | 0.039 | 0.065 | 0.118 |

Non-alcoholic fatty liver disease (NAFLD), Non-alcoholic steatohepatitis (NASH).
